# Flagellin From *Pseudomonas Aeruginosa* Stimulates ATB^0,+^ Transporter for Arginine and Neutral Amino Acids in Human Airway Epithelial Cells

**DOI:** 10.3389/fimmu.2021.641563

**Published:** 2021-03-25

**Authors:** Amelia Barilli, Rossana Visigalli, Francesca Ferrari, Giuseppe Borsani, Valeria Dall'Asta, Bianca Maria Rotoli

**Affiliations:** ^1^Laboratory of General Pathology, Department of Medicine and Surgery, University of Parma, Parma, Italy; ^2^Section of Biology and Genetics, Department of Molecular and Translational Medicine, University of Brescia, Brescia, Italy

**Keywords:** SLC6A14 (ATB^0,+^), glutamine, inflammatory cytokines, inflammasome, NF-kappa B

## Abstract

At present, the central role played by arginine in the modulation of the inflammatory cellular responses is well-recognized, and many pro-inflammatory stimuli are known to modulate the expression and activity of its transmembrane transporters. In this regard, we have addressed the effects of bacterial flagellin from *Pseudomonas aeruginosa* (FLA-PA) on the uptake of the amino acid in human epithelial respiratory cells. Among the arginine transporters, only ATB^0,+^, y^+^L, and y^+^ were operative in bronchial epithelial Calu-3 cells under control conditions; however, only the expression and activity of ATB^0,+^ were stimulated upon incubation with flagellin, whereas those of systems y^+^L and y^+^ were not stimulated. As a result, this induction, in turn, led to an increase in the intracellular content of arginine without making any change to its metabolic pathway. In addition, flagellin upregulated the amount of other amino acids substrates of ATB^0,+^, in particular, all the essential amino acids, such as valine, isoleucine, and leucine, along with the non-essential glutamine. At the molecular level, these effects were directly referable to the stimulation of a toll-like receptor-5 (TLR5) signaling pathway and to the induction of nuclear factor-κB (NF-κB) transcription factor. An induction of ATB^0,+^ expression has been observed also in EpiAirway™, a model of primary human normal tracheal-bronchial epithelial cells that mimics the *in vitro* pseudostratified columnar epithelium of the airways. In this tissue model, the incubation with flagellin is associated with the upregulation of messenger RNAs (mRNAs) for the chemokine IL-8 and for the cytokines IL-6 and interleukin-1β (IL-1β); as for the latter, a marked secretion in the extracellular medium was also observed due to the concomitant activation of caspase-1. The overall findings indicate that, in human respiratory epithelium, flagellin promotes cellular responses associating the increase of intracellular amino acids through ATB^0,+^ with the activation of the inflammasome. Given the role of the ATB^0,+^ transporter as a delivery system for bronchodilators in human airway epithelial cells, its induction under inflammatory conditions gains particular relevance in the field of respiratory pharmacology.

## Introduction

Arginine is a semi-essential amino acid acting as a key driver of metabolic processes associated with several pathological and physiological conditions (1). Specifically, an increasing number of evidence ascribe to a key role of arginine metabolism in the modulation of immune cell functions (2), and arginine is considered as the obligatory substrate of two metabolic pathways central to the inflammatory processes, i.e., the degradation by arginase to ornithine and urea and the conversion to nitric oxide (NO) by nitric oxide synthase (NOS) (3).

In mammalian cells, arginine crosses the plasma membrane through four distinct transport systems: y^+^, y^+^L, b^0,+^, and B^0,+^ [for review see Deves and Boyd (4), Closs et al. (5)]. The cationic amino acid-specific system y^+^ mediates a membrane potential-sensitive, Na^+^-independent arginine transport. The activity of system y^+^ is referable to the cationic amino acid transporter (CAT) family of monomeric transporters, which includes the ubiquitous *SLC7A1*-encoded CAT1 and the two transporters, CAT2A and CAT2B, derived from the alternative splicing of a *SLC7A2* transcript. Conversely, system y^+^L mediates a Na^+^-independent transport of arginine and a Na^+^-dependent transport of neutral amino acids, particularly leucine. Under physiological conditions, it operates as an antiport by exchanging intracellular arginine with extracellular neutral amino acids and sodium. System y^+^L belongs to the group of the heterodimeric amino acid transporters (HAT) and is formed by the heavy subunit 4F2hc/CD98 and one of the two alternative light chains, y^+^LAT1 (encoded by *SLC7A7*) or y^+^LAT2 (encoded by *SLC7A6*) (6). Also, system b^0,+^ belongs to the group of the HAT and is formed by b^0,+^AT (encoded by *SLC7A9*) linked through a disulfide bridge to the heavy subunit rBAT. This system is expressed in the small intestine and the proximal tubule of the kidney, where it mediates the reabsorption of cystine by exchanging extracellular dibasic amino acids and cystine with intracellular neutral amino acids (7). Another transporter involved in the arginine absorption is ATB^0,+^, an influx system for neutral and cationic amino acids endowed with a high concentrative capacity, which is energized by the transmembrane gradients of Na^+^ and Cl^−^ as well as by the membrane potential (8). This transporter, first described in human epithelial airway cells by Galietta et al. (9), is mainly expressed in the lung and the intestine (10, 11). In *in vitro* models, only some airway epithelial cells, such as Calu-3 and NCl-H441, actually express ATB^0,+^, while other cells, such as A549 and BEAS-2B cells, do not (12). Recently, we have demonstrated that *SLC6A14*/ATB^0,+^ is maximally expressed on the apical side of EpiAirway™, a model of primary human tracheal-bronchial epithelial cells where it mediates the uptake of carnitine (13).

Most of intracellular arginine is known to depend on the uptake from the extracellular milieu, since a *de novo* synthesis is not sufficient to meet the metabolic needs under particular conditions, such as infections or inflammation (14). Consistently, pro-inflammatory mediators, such as lypolisaccaride (LPS), tumor necrosis factor-α (TNFα), interleukin-1β (IL-1β), and interferon-γ (IFNγ), have been described over the past few years, to enhance the cellular uptake of arginine through an increased expression of CAT transporters in a variety of cell models, mainly immune and endothelial cells (15–20). More recently, also the ATB^0,+^ activity in bronchial epithelial cells has been reported to be modulated by inflammatory stimuli, such as LPS and TNFα (13), and flagellin from *Pseudomonas aeruginosa* (FLA-PA) (21). In this study, we further explored the effects of flagellin on human bronchial cells by extending the study to all arginine transporters as well as by addressing the molecular pathway involved in the stimulation of *SLC6A14*/ATB^0,+^. Moreover, we also evaluated the activation of inflammasome as a consequence of flagellin-mediated induction of ATB^0,+^ in normal tracheal-bronchial EpiAirway™ tissues.

## Materials and Methods

### Cell Culture and Experimental Treatments

Calu-3 cells (American Type Culture Collection, VA, USA), obtained from a human lung adenocarcinoma and derived from serous cells of proximal bronchial airways, were routinely grown in 10-cm diameter dishes in Eagle's Minimum Essential Medium (EMEM) supplemented with 10% fetal bovine serum (FBS), sodium pyruvate (1 mM), and 1% penicillin/streptomycin. Cells between passages 25 and 32 were routinely maintained under physiological conditions (37.5°C, 5% CO_2_, 95% humidity). EpiAirway™ tissues (AIR-100), an organotypic *in vitro* model of primary human tracheal-bronchial epithelial cells that form a fully differentiated, pseudostratified columnar epithelium, were provided by MatTek IVLSL (Bratislava, Slovakia) and were cultured by following the instructions of the manufacturer as described in a previous study (22).

The experimental treatment was performed by adding a purified FLA-PA (Invivogen, CA, USA) to complete growth medium; for EpiAirway™, FLA-PA was added to the apical compartment. Nuclear factor-κB (NF-κB) inhibitors were added to the cell culture 1 h before the addition of flagellin when employed. No significant cell loss was observed for up to 24 h under any of the experimental conditions.

### Real-Time Quantitative PCR Analysis

Gene expression has been analyzed through real-time quantitative PCR (RT-qPCR) as described in a previous study (23). About 1 μg of complementary DNA (cDNA) was obtained upon a reverse transcription of total RNA with the RevertAid First Strand cDNA Synthesis Kit (ThermoFisher Scientific, MA, USA); the qPCR analysis was then performed on a StepOnePlus Real-Time PCR System (ThermoFisher Scientific, MA, USA) by employing specific forward/reverse primer pairs (Table 1) and SYBR™ Green or TaqMan Gene Expression Master Mix (ThermoFisher Scientific, MA, USA). The expression of the gene of interest under each experimental condition was calculated by using the standard curve method (24) after normalization of the housekeeping gene (Ribosomal like protein 15, *RPL15*, Gene ID:6138).

**Table 1:** Sequences of the primer pairs employed for real-time quantitative PCR (RT-qPCR) analysis.

**Gene/protein name (Gene ID)**	**Forward primer**	**Reverse primer**
*ARG2/*Arginase2 (ID: 384)	AAGCTGGCTTGATGAAAAGGC	GCGTGGATTCACTATCAGGTTGT
*NOS3/*eNOS (ID: 4846)	TGGTACATGAGCACTGAGATCG	CCACGTTGATTTCCACTGCTG
*IL1B/*IL-1β (ID: 3553)	Hs99999029_m1 (TaqMan^®^ Assay, ThermoFisher Scientific)	
*IL6/*IL-6 (ID: 3569)	AACCTGAACCTTCCAAAGATGG	TCTGGCTTGTTCCTCACTACT
*SLC1A5/*IL-8 (ID: 3576)	ACTGAGAGTGATTGAGAGTGGAC	AACCCTCTGCACCCAGTTTTC
*SLC6A14/*ATB^0,+^ (ID: 11254)	GCTGCTTGGTTTTGTTTCTCCTTGGTC	GCAATTAAAATGCCCCATCCAGCAC
*SLC7A1/*CAT1 (ID: 6541)	CTTCATCACCGGCTGGAACT	GGGTCTGCCTATCAGCTCGT
*SLC7A2/*CAT2A (ID: 6542)	TTCTCTCTGCGCCTTGTCAA	TCTAAACAGTAAGCCATCCCGG
*SLC7A2/*CAT2B (ID: 6542)	TTCTCTCTGCGCCTTGTCAA	CCATCCTCCGCCATAGCATA
*SLC7A6/*y+LAT1 (ID: 9057)	Hs00187757_m1 (TaqMan^®^ Assay, ThermoFisher Scientific)	
*SLC7A7/*y+LAT2 (ID: 9056)	Hs00909952_m1 (TaqMan^®^ Assay, ThermoFisher Scientific)	
*TLR5/*TLR5 (ID: 7100)	TCCCTGAACTCACGAGTCTTT	GGTTGTCAAGTCCGTAAAATGC
*RPL15/*RPL15 (ID: 6138)	GCAGCCATCAGGTAAGCCAAG	AGCGGACCCTCAGAAGAAAGC

### *SLC6A14* Promoter Sequence Analysis

The analysis of a *SLC6A14* promoter sequence was performed by employing the tools available online^1^ The sequence upstream of the transcription start site (TSS) of the *SLC6A14* gene was obtained with a Sequence Retrieval Tool available from The Eukaryotic Promoter Database (25). Then, the Search Motif Tool was sed to scan the promoter region with position weight matrices (PWM) of NF-κB transcription factors obtained from the open-source JASPAR database (26). The scan was performed on the fly using the FindM tool from the Signal Search Analysis toolkit (27) at the Swiss Institute of Bioinformatics.

### Western Blot Analysis

For the determination of ATB^0,+^ expression, cells were lysed in a RIPA buffer added with a cocktail of protease inhibitors (Complete, Mini, EDTA-free, Roche). The Western Blot analysis was performed as described in a previous study (28). Briefly, 20 μg of proteins were separated on the sodium dodecyl sulfate-polyacrylamide gel electrophoresis (SDS-PAGE) (4–12% acrylamide) and were electrophoretically transferred to polyvinylidene fluoride (PVDF) membranes (Immobilione-P membrane, Merck, NJ, USA). Membranes were first incubated for 1 h at room temperature (RT) in a Tris-buffered saline solution (TBS; 50 mM Tris-HCl pH 7.5, 150 mM NaCl) containing 5% non-fat dried milk and then incubated overnight at 4°C in a Tris-buffered saline with Tween 20 (TBST) buffer added with 5% bovine serum albumin (BSA) and anti-ATB^0,+^ purified rabbit polyclonal antibody (1:5,000, Merck, NJ, USA), anti-p65 NF-κB, or anti-phospho-p65 (Ser536) NF-κB (1:2000, Cell Signaling TECHNOLOGY, MA, USA). Vinculin, detected with a monoclonal antibody (1:2,000; Merck, NJ, USA), was employed as internal standard. Immunoreactivity was visualized by using the Immobilon Western Chemiluminescent HRP Substrate (Merck, NJ, UA). Western Blot images were captured by using an iBright FL1500 Imaging System (ThermoFisher Scientific, MA, USA) and analyzed with the iBright Analysis Software.

### Amino Acid Uptake

For transport studies, Calu-3 cells were cultured onto 96-well trays (Falcon). After two rapid washes in a pre-warmed transport buffer [Earle's Balanced Salt Solution (EBSS) containing (in mM) 117 NaCl, 1.8 CaCl_2_, 5.3 KCl, 0.9 NaH_2_PO_4_, 0.8 MgSO_4_, 5.5 glucose, 26 Tris/HCl, adjusted to pH 7.4], the cells were incubated for 30 s in the same solution containing [^3^H]arginine (50 μM, 5 μCi/ml) in the absence or presence of the indicated amino acids (2 mM each) employed as inhibitors. When sodium-independent transport was measured, a modified Na^+^-free EBSS (NMG-EBSS) was employed, with NaCl and NaH_2_PO_4_ replaced with N-methyl-D-glucamine and choline salts, respectively. The experiment was terminated by two rapid washes (<10 s) in an ice-cold 300 mM urea. Intracellular radioactivity from the cell monolayers was extracted in ethanol and measured by using the MicroBeta^2®^ liquid scintillation spectrometer (PerkinElmer, MA, USA). The uptake was normalized for the protein content, determined directly in each well by using a modified Lowry procedure (29), and expressed as nmol/mg of protein/min.

### Determination of the Amino Acid Intracellular Content

For the measurement of intracellular amino acids, Calu-3 cells, grown on 24-well trays were rapidly washed with an ice-cold PBS, and the intracellular pool was extracted with a 10-min incubation in 200 μl of ethanol at 4°C. After freeze-drying, samples were suspended in 150 μl Lithium Loading Buffer (Biochrom, Cambridge, UK), and the intracellular content of each amino acid species was determined through a high-pressure liquid chromatography (HPLC) analysis with a Biochrom 30 Amino Acid Analyzer (Biochrom, Cambridge, UK), employing a high-resolution lithium column and lithium buffers for elution (Biochrom, Cambridge, UK). The column effluent was mixed with an EZ Nin Reagent Kit (Biochrom, Cambridge, UK), passed through the high-temperature reaction coil, and read by the photometer unit at both 570 and 440 nm. Protein content in each condition was determined using a modified Lowry procedure (29), and the content of amino acids was expressed as nmol/mg of protein.

### Interleukin-1β Measurement

Supernatants collected from the FLA-PA-treated EpiAirway™ tissues were assayed for secreted IL-1β using the Human IL-1 beta/IL-1F2 Quantikine ELISA Kit (R&D) according to the instructions of the manufacturer. Briefly, cell supernatants were added to the plate along with blank and standard samples. After 2 h incubation at RT, samples were first incubated for 1 h with Human IL-1β Conjugate and then with the substrate solution for further 20 min. In the end, the reaction was stopped with the stop solution, and the optical density of each well was read by using an EnSpire® Multimode Plate Reader (Perkin Elmer, MA, USA), at 450 nm. The amount of the cytokine in each sample was calculated from the standard curve and expressed as pg/ml of the incubation medium.

### Caspase-1 Activity

The activity of caspase-1 was measured with a CaspaseGlo®-1 Inflammasome Assay (Promega, WI, USA) in the incubation medium of FLA-PA-treated EpiAirway™ tissues according to the instructions of the manufacturer. Briefly, 50 μl of cell supernatant or fresh growth medium (blank) was transferred into a 96-well plate and mixed with 50 μl of a CaspaseGlo®-1 Reagent, previously completed with a 6 μl/ml MG132 inhibitor. After the incubation at RT for 1 h, luminescence was read with the EnSpire® Multimode Plate Reader (Perkin Elmer, MA, USA); a blank value was subtracted from the signals measured in each well.

### Statistical Analysis

GraphPad Prism 7 (GraphPad Software) was used for statistical analysis. The values of *p* were calculated with a two-tailed Student's *t*-test; *p* < 0.05 was considered significant.

### Materials

Fetal bovine serum was purchased from EuroClone (Italy). L-[2,3,4-^3^H]-monohydrochloride Arginine (54.5 mCi/mmol) was obtained from PerkinElmer. Caffeic acid phenethyl ester (CAPE) was from Calbiochem (Italy) while all other chemicals were from Merck (Italy).

## Results

**Figure 1:**
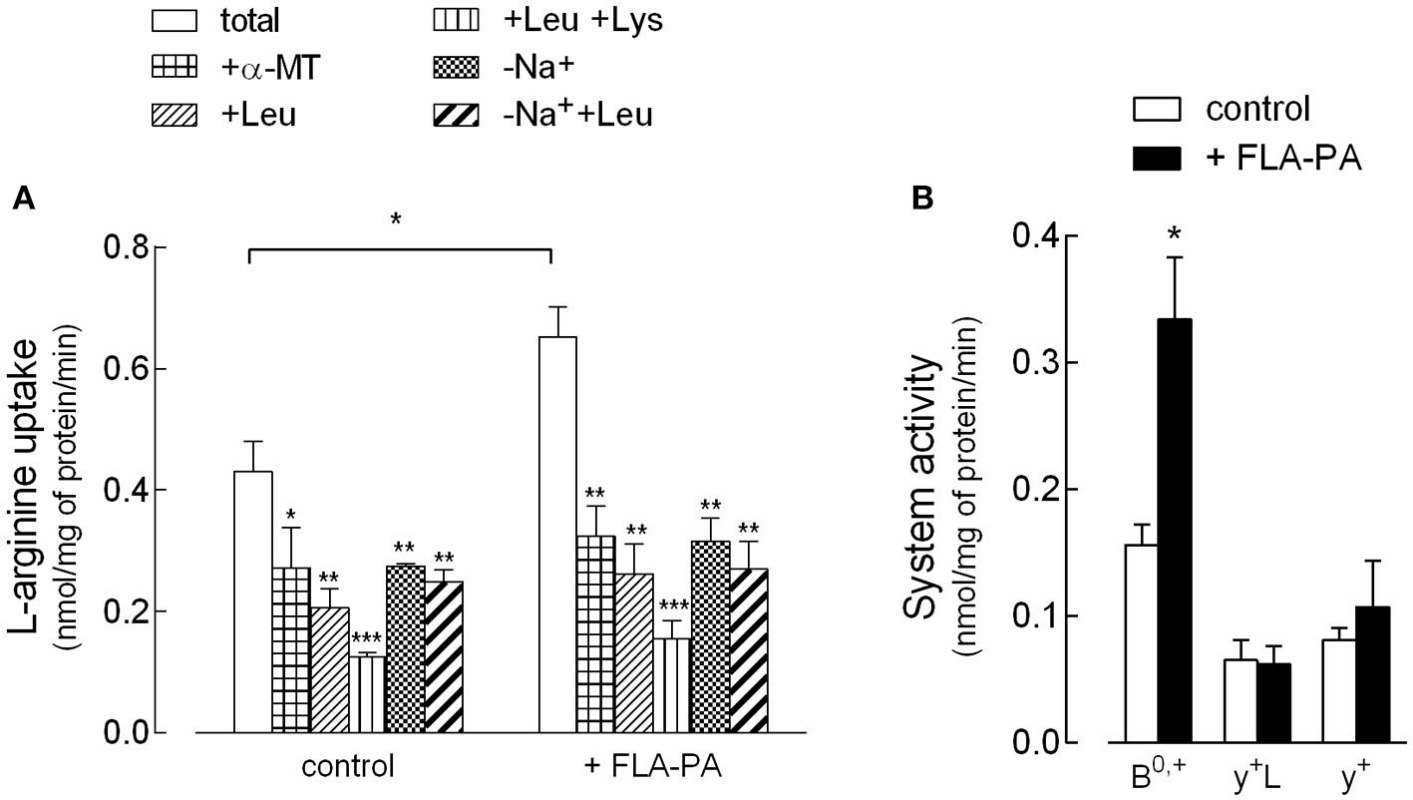
Calu-3 cells were incubated for 8 h in the absence (control) or in the presence of 2 μg/ml flagellin from *Psuudomonas aeruginosa* (FLA-PA). **(A)** 30 s-[^3^H]arginine uptake (50 μM; 5 μCi/ml) was measured both in Na^+^-containing (total uptake) and Na^+^-free (-Na^+^) Earle's Balanced Salt Solution (EBSS), in the absence or in the presence of 2 mM methyltryptophan (α-MT), leucine, or leucine and lysine, as indicated. Bars represent the mean ± SEM of three independent determinations. *p < 0.05, **p < 0.01, ***p < 0.001 vs. total. **(B)** Data of **(A)** were employed to calculate the contribution of systems ATB^0,+^, y^+^L, and y^+^ to total uptake as follows: ATB^0,+^ = Na^+^-dependent uptake; y+L= “+Leu uptake” – “+α-MT uptake”; y^+^ = “+Leu uptake” – “+Leu+Lys uptake”. *p < 0.05 vs. control.

First, the effect of FLA-PA on the activity and expression of arginine transporters in Calu-3 bronchial epithelial cells has been investigated. In this regard, experimental conditions were adopted, which allowed the discrimination of the contribution of the different arginine transporters to the total uptake (Figure 1). Specifically, the presence/absence of Na^+^ was employed to calculate the activity of ATB^0,+^ as the sodium-dependent fraction of the transport, while further addition of leucine, α-methyltryptophan (α-MT), and lysine allowed the evaluation of the activity of systems y^+^L, y^+^, and b^0,+^. In the presence of Na^+^, leucine is known to inhibit both ATB^0,+^ and y^+^L transporters, while α-MT only inhibits ATB^0,+^ (30). As a result, the contribution of y^+^L can be obtained by subtracting the component inhibited by α-MT from the component inhibited by leucine. Second, since further addition of lysine also inhibits system y^+^, system y^+^ can be calculated as the difference between the uptake measured in the presence of leucine and the uptake measured in the presence of both leucine and lysine. Finally, the uptake mediated by b^0,+^ can be estimated as the quote inhibited by leucine in the absence of sodium. Figure 1A presents the results of the arginine uptake measured under these experimental conditions, both in control, untreated cells, and upon the incubation for 8 h with 2 μg/ml FLA-PA. These data have then been used to calculate the contribution of the different transport systems to the total uptake (Figure 1B). The results, besides excluding the presence of system b^0,+^ in Calu-3 cells, clearly demonstrated the activity of systems ATB^0,+^, y^+^L, and y^+^ under control conditions. Upon incubation with FLA-PA, a significant increase of total arginine uptake was observed and was referable to the induction of the activity of an ATB^0,+^ transporter, while neither system y^+^ nor system y^+^L was modified by the experimental treatment. Interestingly, the induction of ATB^0,+^ by flagellin was comparable when calculated as the sodium-dependent fraction of arginine uptake or, rather, as the α-MT-inhibited quote.

**Figure 2:**
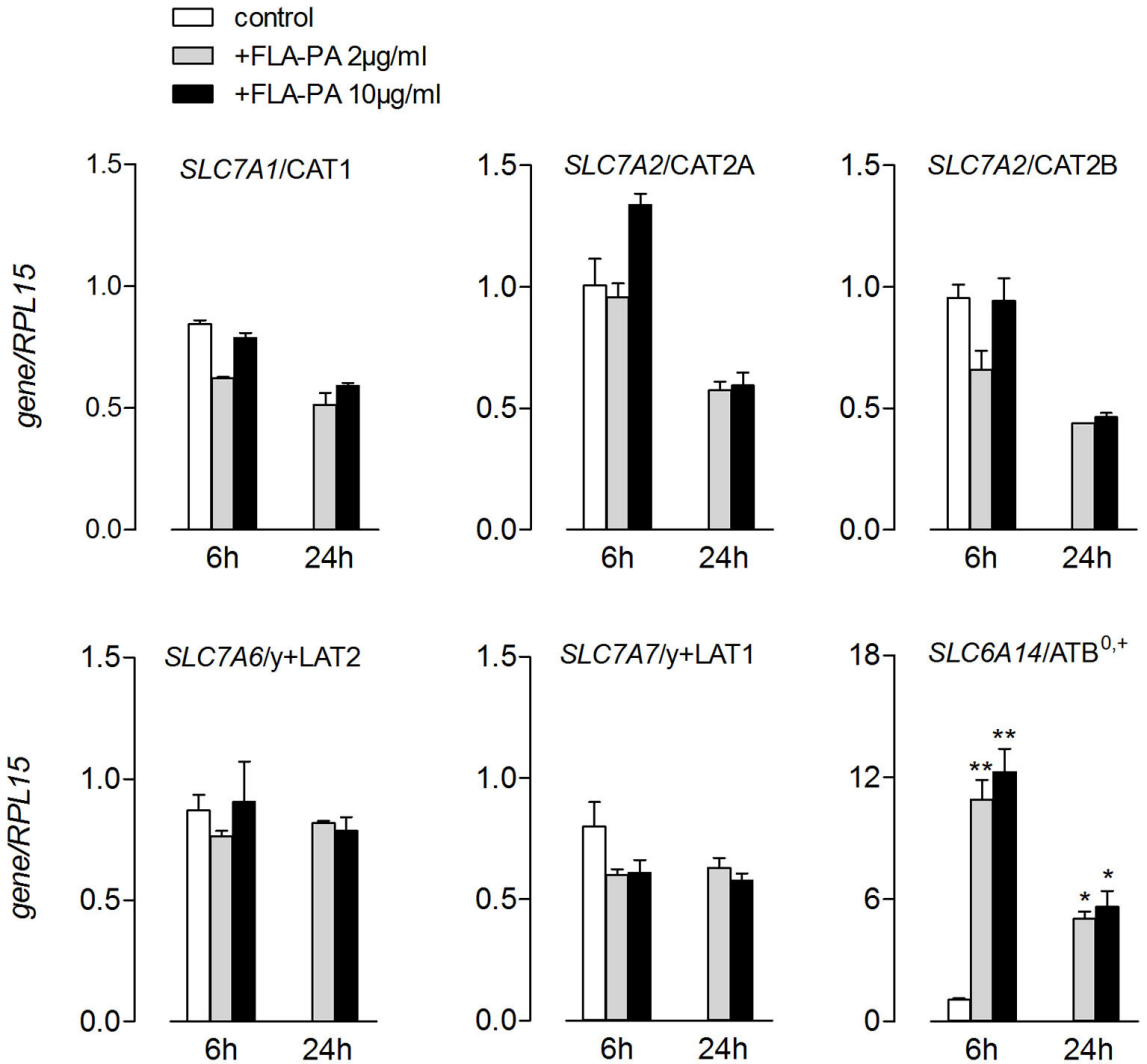
Calu-3 were incubated for 6 and 24 h in the presence of the indicated concentrations of FLA-PA; the expression of the indicated genes was then measured by means of real-time quantitative PCR (RT-qPCR) and normalized for the housekeeping gene *(RPL15)*. Data of three experiments, each performed in duplicate, are presented as mean ± SEM. *p < 0.05, **p < 0.01 vs. control.

The transport data were in line with those of the gene expression (Figure 2). Indeed, the treatment with FLA-PA induced a marked increase of *SLC6A14* mRNA, coding for a ATB^0,+^ transporter, without affecting the expression of neither system y^+^- (*SLC7A1*/CAT1, *SLC7A2/*CAT2A, and *SLC7A2/*CAT2B) nor system y^+^L- (*SLC7A7*/y+LAT1 and *SLC7A6*/y+LAT2) related genes. The same pattern of expression was observed when higher concentrations of flagellin (10 μg/ml) were employed or when the incubation time prolonged from 6 to 24 h. Altogether, these findings pointed to ATB^0,+^ as the sole arginine transporter targeted by flagellin in Calu-3 cells.

**Figure 3:**
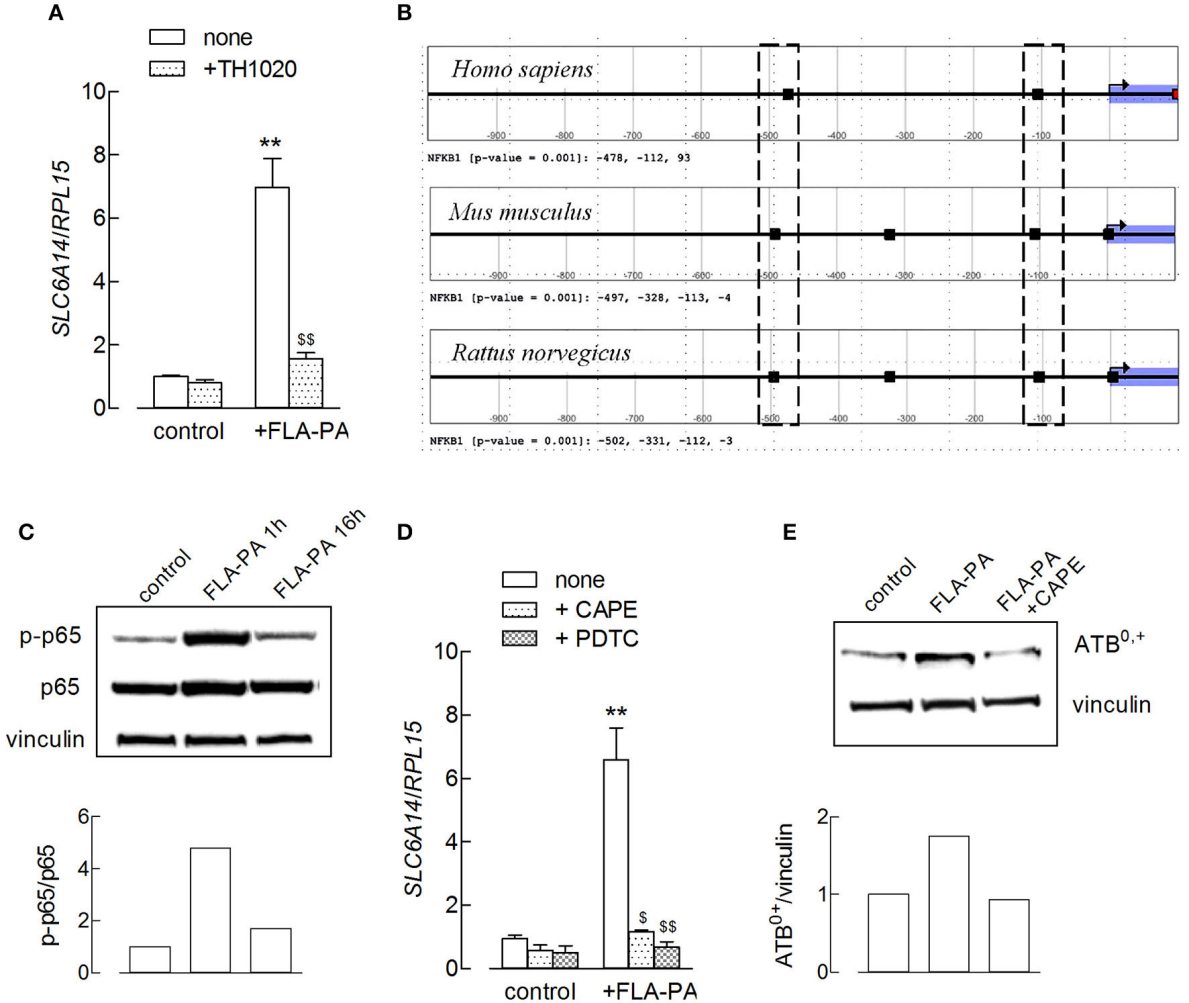
**(A)** Calu-3 cells were pretreated for 1 h with TH1020 (20 μM), before the incubation with 2 μg/ml FLA-PA for 6 h. The expression of *SLC6A14*/ATB^0,+^ was then determined by means of RT-qPCR, as described in the section “Materials and methods”. Data of three experiments, each performed in duplicate, are presented as mean ± SEM. **p < 0.01 vs. control, ^$$^*p* < 0.01 vs. none + FLA-PA. **(B)** Output of the Search Motif Tools scan of human, mouse, and rat *SLC6A14* promoters for the nuclear factor-κB (NF-κB)1 position weight matrix profile MA0105.4 from Jaspar. The analysis has been performed in a region from −1,000 to +100 relative to transcription start site (TSS) and with the default cutoff (*p*-value) of 0.001. Hits are marked as black rectangles in the plot, and exact positions relative to the TSS are reported below each plot. The dotted boxes evidence NF-κB1 motifs located in equivalent positions in the promoter of the three mammalian species. **(C)** The amount of p-p65 and p65 proteins was determined by using the Western Blot analysis (see section “Material and methods”) in Calu-3 cells treated with 2 μg/ml FLA-PA; the densitometric analysis of protein expression is shown. A representative blot is reported that was repeated three times, showing comparable results. **(D)** Calu-3 cells were pretreated for 1 h with caffeic acid phenethyl ester (CAPE) (25 μg/ml) or pyrrolidine dithiocarbamate (PDTC) (100 μM) before the incubation with 2 μg/ml FLA-PA for 6 h. The expression of *SLC6A14*/ATB^0,+^ was then determined by means of RT-qPCR as described in section “Materials and methods”. Data of three experiments are presented as mean ± SEM, each performed in duplicate. **p < 0.01 vs. control, ^$^*p* < 0.05, ^$$^*p* < 0.01 vs. none + FLA-PA. **(E)** The amount of ATB^0,+^ protein was determined by using the Western Blot analysis (see section “Material and methods”) in Calu-3 cells treated for 8 h with 2 μg/ml FLA-PA, both in the absence and in the presence of CAPE; the densitometric analysis of protein expression normalized for that of vinculin is shown. A representative blot of three replicates that gave comparable results is shown.

Flagellin is known to exert its pro-inflammatory effects through the activation of NF-κB *via* the stimulation of a Toll-like receptor-5 (TLR5) receptor (31, 32). Actually, the use of TLR5-agonist TH1020 completely prevented the flagellin-induced increase of *SLC6A14*/ATB^0,+^ expression (Figure 3A). To investigate the role of a NF-κB transcription factor, a bioinformatic analysis was first carried out on the promoter sequence of the *SLC6A14* gene to identify the presence of putative NF-κB binding sites. NF-κB is a family of dimeric DNA binding transcription factors; five related protein subunits are known in mammals, NF-κB1/p50, NF-κB2/p52, RelA/p65, RelB, and c-Rel. Each family member shares a high amino acid sequence identity throughout a region of about 300 amino acids located near their NH_2_-termini and referred to as the Rel Homology Region (RHR). RHR is responsible for sequence-specific DNA binding, protein dimerization, and the association with a class of NF-κB inhibitor proteins known as IkB (33). The best-studied κB sites fit the consensus site pattern 5′-GGGRNWYYCC-3′ (where R, W, Y, and N denote purine, adenine or thymine, pyrimidine, and any nucleotide, respectively), although NF-κB can bind to degenerate κB sites (34). The analysis carried out in this study shows that the human *SLC6A14* promoter region (from −1,000 to +100 relative to the transcriptional start site) contains three NF-κB1 motifs. A comparative promoter analysis revealed that the mouse and rat *Slc6a14* promoter sequences present two NF-κB1 motifs in the positions equivalent to those of the human promoter, supporting an evolutionarily conserved regulatory mechanism (Figure 3B). These data suggest a direct involvement of NF-κB transcription factors in the modulation of *SLC6A14* expression. In line with these findings, we show here that the incubation of Calu-3 cells with FLA-PA determined early, transient phosphorylation of NF-κB p65 subunit at Ser536 (Figure 3C), indicating the activation of the transcription factor under our experimental conditions. Moreover, the preincubation of cells with NF-κB inhibitors, such as CAPE or pyrrolidine dithiocarbamate (PDTC), completely prevented the effect of flagellin on *SLC6A14* expression (Figure 3D); the addition of CAPE hampered the induction also at the protein level (Figure 3E). Hence, we can conclude that the induction of the *SLC6A14* gene by flagellin directly depends upon the activity of the TLR5/NF-κB signaling pathway.

**Figure 4:**
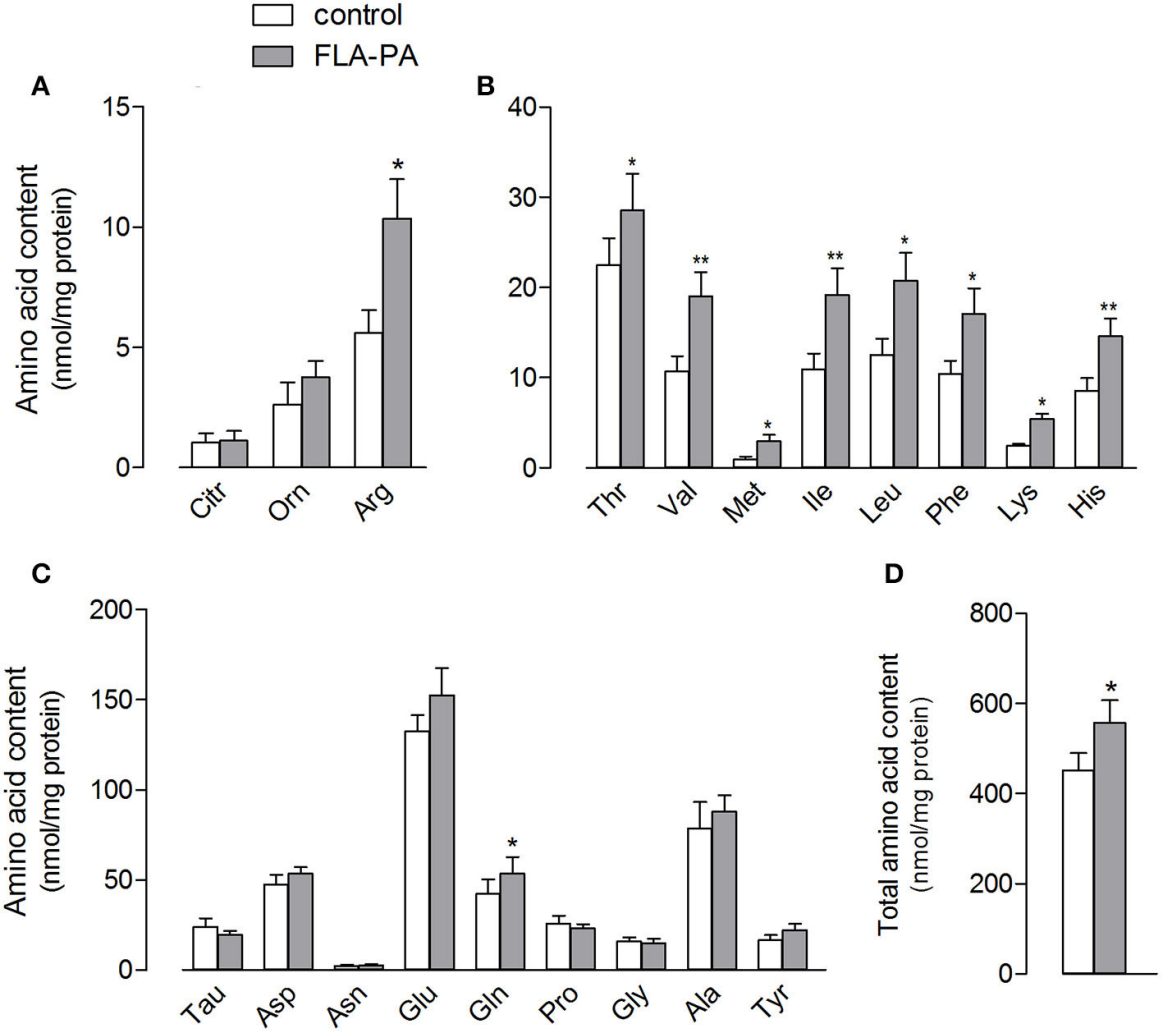
Calu-3 cells were treated for 24 h with 2 μg/ml FLA-PA and the intracellular content of arginine and its metabolites **(A)**, essential **(B)**, and non-essential **(C)** amino acids was determined as described in section “Materials and methods”. Total amino acid content is also shown **(D)**. Bars represent the mean ± SEM of four independent experiments. *p < 0.05, **p < 0.01 vs. control.

The stimulation of the ATB^0,+^ expression and activity by FLA-PA was also associated to changes in the intracellular amino acid content of Calu-3 cells (Figure 4), which increased from 451.9 ± 37.8 to 556.3 ± 50.8 nmol/mg of protein (Figure 4D). Specifically, in line with the changes observed in the arginine uptake, the intracellular amount of this amino acid nearly doubled in treated cells; however, this increase was not associated with any change in the content of its metabolites, citrulline and ornithine (Figure 4A). All essential amino acids, such as valine, isoleucine, and leucine, were significantly increased in the cells incubated with FLA-PA (Figure 4B), while almost all non-essential amino acids remained unaffected except glutamine (Figure 4C).

**Figure 5:**
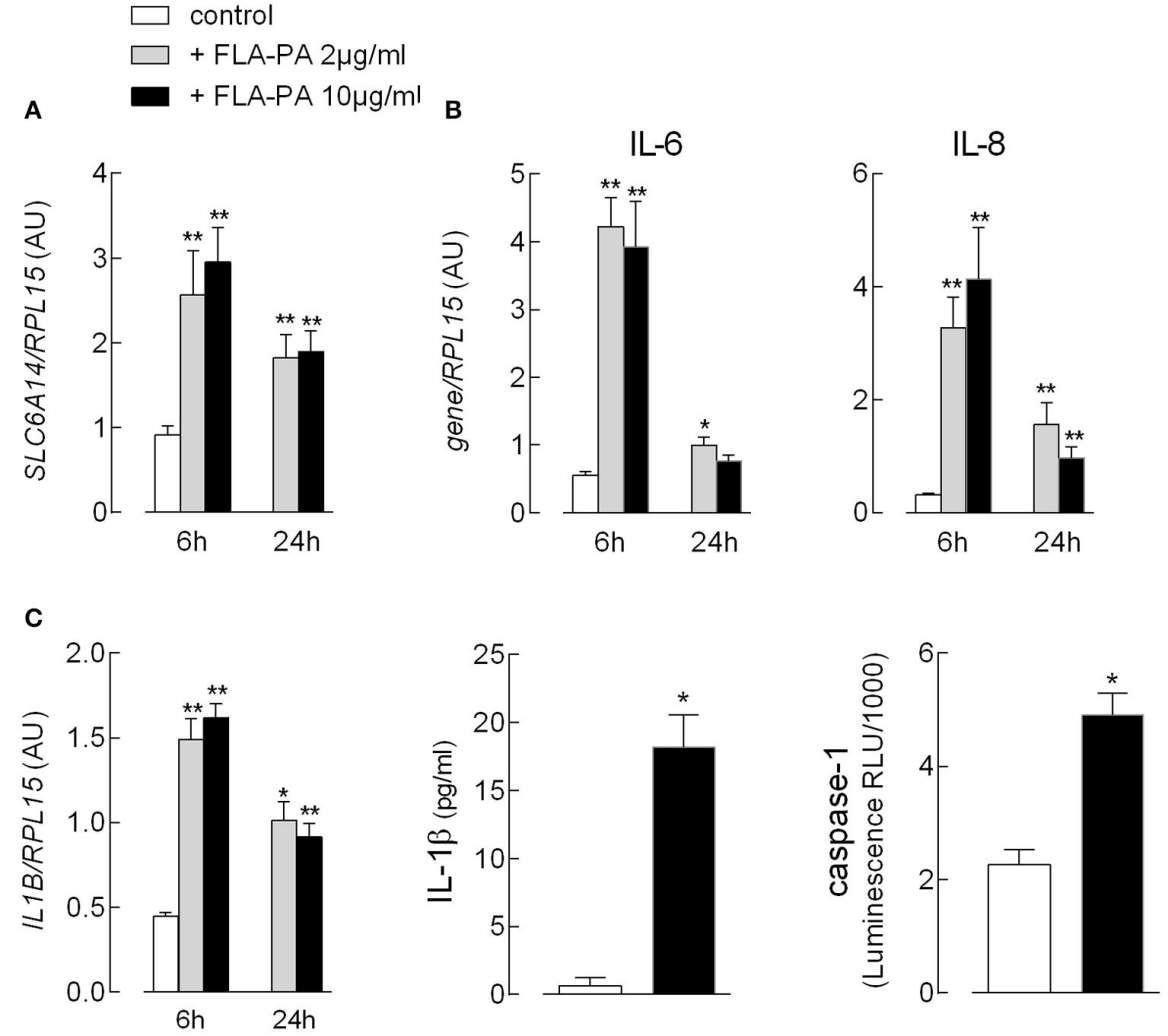
EpiAirway™ tissues were incubated in the absence (control) or in the presence of the indicated concentrations of FLA-PA. After 6 h, the expression of the indicated genes **(A,B)** was measured by means of RT-qPCR analysis and shown after normalization for that of the housekeeping gene *(RPL15)*. Data of three experiments, each performed in duplicate, are presented as mean ± SEM. **(C)** The activation of inflammasome was monitored after 16 h by addressing the expression of interleukin-1β (IL-1β) mRNA (left) and the cytokine secretion (middle) with RT-qPCR and ELISA assay, respectively; the activity of caspase-1 (right) was measured in parallel, as described in section “Materials and methods”. Bars represent the mean ± SEM of three experiments. *p < 0.05, **p < 0.01 vs. control.

Since we recently demonstrated that EpiAirway™, a cellular model of normal human bronchial epithelium, maximally expresses *SLC6A14* on the apical membrane (35), we next addressed the effect of FLA-PA in this model. First, we evaluated the expression of flagellin-targeted TLR5 and found it to be readily detectable and more abundant than that in Calu-3 cells and monocyte-derived macrophages (MDM) (Supplementary Figure 1). Similar to Calu-3 cells, the exposure of EpiAirway™ to bacterial flagellin caused a significant increase in the expression of ATB^0,+^ transporter (Figure 5A). Under the same condition, a marked induction of the pro-inflammatory phenotype was also observed, as indicated by the transient increase of mRNAs coding for the chemokine IL-8, as well as for the cytokines IL-6 (Figure 5B) and IL-1β (Figure 5C). For the cytokine IL-1β, it is important to notice that the incubation with flagellin was also associated with a markedly increased secretion of the cytokine in the extracellular medium, likely due to the concomitant activation of caspase-1 observed under the same experimental condition.

## Discussion

An increasing number of evidence in the past decade has highlighted the role of solute carrier (SLC) transporters in shaping the immune responses by modulating the bioavailability of many metabolites, including amino acids and their derivatives (36). In this context we demonstrate here that, in Calu-3 bronchial epithelial cells and EpiAirway™ tissues, the induction of a pro-inflammatory phenotype by bacterial FLA-PA is associated with an upregulation of the expression and activity of *SLC6A14*/ATB^0,+^ transporter for cationic and neutral amino acids.

ATB^0,+^ is a highly concentrative electrogenic transport system accepting all essential amino acids, along with arginine and glutamine (37). Recent studies identified *SLC6A14* as a modifier gene in cystic fibrosis (CF), where its genetic variants modulate the severity of pulmonary disease (21, 38). In this context, the arginine transport through ATB^0,+^ has been shown to increase the residual function of F508del-CFTR chloride channel, suggesting a positive functional interaction between the CF modifier SLC6A14 and CFTR protein (39). The expression of *SLC6A14*/ATB^0,+^ is normally low under physiological conditions, while it is significantly induced in tumors like colon, cervical cancer, and breast cancer where its high activity is supposed to meet the increasing demand for amino acids essential for the rapid growth of the tumor (40). Similarly, ATB^0,+^ has been found upregulated also under intestinal inflammatory states, such as ulcerative colitis and Crohn's disease (41). As for the respiratory epithelium, we have recently demonstrated an induction of *SLC6A14*/ATB^0,+^ by LPS and TNF-α in Calu-3 and EpiAirway™ cells, in which the presence of the transporter was high and confined to the apical membrane (13).

In the same models, we demonstrate here that the expression of *SLC6A14*/ATB^0,+^ is also stimulated by FLA-PA and that this, in turn, causes a significant increase of the intracellular content of all essential amino acids, such as valine, phenylalanine, leucine, and isoleucine, along with the semi-essential arginine and non-essential glutamine, amino acids that are recognized as substrates of the transporter (40). The role of this amino acid upregulation has yet to be clarified. As for arginine, the immunomodulatory properties of the amino acid and its metabolites are well-recognized (42). Arginine is indeed the obligatory substrate for the synthesis of mediators crucial for the inflammatory response: NO, involved in processes associated with vasodilatation and cytotoxicity, which is produced by the NOS, and ornithine and urea, precursors for the production of polyamines and proline, generated by arginase (43). In our hands, however, the increased amount of intracellular arginine is not associated with a change of its metabolism, since neither citrulline, a byproduct of NO production, nor ornithine levels are modified in FLA-PA-treated cells, thus excluding the activation of either metabolic pathways. No change is consistently observed in the expression of NOS or arginase enzymes or in the NO synthesis as determined through the nitrite production in the cultured medium (data not shown). On the other hand, it is known that the intracellular concentration of the amino acid is not limiting for NOS (14), and the NO production in different cell models mostly depends upon the uptake of extracellular arginine by CAT transporters (18, 44, 45). Since we demonstrate in this study that the expression and activity of arginine transporters other than ATB^0,+^ do not change upon the incubation of Calu-3 cells with FLA-PA, we can conclude that the stimulation of ATB^0,+^ activity by flagellin only causes an increase of intracellular arginine availability without affecting its metabolism. Di Paola and colleagues have suggested that the induction of *SLC6A14*/ATB^0,+^ by FLA-PA in the airways aims to prevent the attachment of *P. aeruginosa* by depleting extracellular arginine from the airways surface liquid. According to their findings, *SLC6A14* may thus have an important role in the modulation of lung disease in patients with CF, likely through its role in the host defense (21). Whether the relevance of *SLC6A14* is actually restricted to the limitation of extracellular arginine or, rather, has a role in modulating the intracellular content of the amino acid for unrevealed functions remains to be investigated. Really, our results extend the role of ATB^0,+^ as a modulator of the intracellular and extracellular availability, not just of arginine but also of other amino acids, and support the hypothesis, of Broer and Fairweather (41), that the transporter expressed at places where the body interfaces with microbes, such as lung and intestine, may be involved in reducing the availability of nutrients essential to bacteria.

In respiratory and intestinal epithelia, as well as in monocytic cells, extracellular flagellin is known to target TLR5 and triggers a signaling cascade, leading to the activation of a NF-κB transcription factor for the induction of inflammatory genes (31, 46–49); the expression of numerous ATP-binding cassette (ABC) and SLC transporters in animal models has been shown to depend on the activity of the same transcription factor (50). In the present study, we have shown that a consensus domain for NF-κB is present also in the *SLC6A14* gene; moreover, we confirmed that flagellin actually promotes NF-κB p65 phosphorylation and provided evidence that the flagellin-dependent induction of *SLC6A14* requires NF-κB. Since also Signal Transducers and Activators of Transcription (STAT) proteins are described to be involved in the transcription of *SLC6A14* (21), it is conceivable that both the transcription factors contribute to the FLA-PA-driven induction of the transporter. A similar hypothesis has been proposed in the model by Bao et al. for the intracellular signaling pathway primed by *Salmonella typhimurium* flagellin in macrophages (49).

In EpiAirway™, the exposure to FLA-PA not only induced the expression of *SLC6A14* but also caused a massive increase in the expression of the chemokine IL-8, as well as of the cytokines IL-6 and IL-1β; under the same conditions, also caspase-1 was activated, leading to a large secretion of IL-1β in the extracellular medium, and, hence, showing for the first time the flagellin-dependent activation of inflammasome in EpiAirway™.

In conclusion, our data indicate that flagellin promotes the cellular responses that associate the activation of inflammasome with the upregulation of the intracellular content of amino acids through ATB^0,+^ that may be thus included in the list of genes upregulated by inflammatory stimuli. Since we have recently shown that ATB^0,+^ is a delivery system for bronchodilators in human airway epithelial cells (13), its induction under inflammatory conditions may gain a particular relevance in the field of respiratory pharmacokinetics.

## Data Availability Statement

The raw data supporting the conclusions of this article will be made available by the authors, without undue reservation.

## Author Contributions

BR and VD designed the experimental plan. FF, AB, and RV performed *in vitro* experiments. GB performed the computational promoter analysis. AB and VD analyzed the results. BR and VD wrote the paper. All authors read and approved the final manuscript.

## Conflict of Interest

The authors declare that the research was conducted in the absence of any commercial or financial relationships that could be construed as a potential conflict of interest.

## Footnotes

^1^The Eukaryotic Promoter Database: https://epd.epfl.ch/; JASPAR database: http://jaspar.genereg.net/; Signal Search Analysis toolkit: https://ccg.epfl.ch/ssa/.

## Supplementary Material

Supplementary Figure 1 The expression of toll-like receptor-5 (TLR5) was measured by means of RT-qPCR analysis and shown after normalization for that of the housekeeping gene *(RPL15)*. Data of the three different cultures are presented as mean ± SEM. Monocyte-derived macrophages (MDM) were obtained from human monocytes after 5 day differentiation in the presence of 50 ng/ml GM-CSF.

## Abbreviations

EBSS: Earle's Balanced Salt Solution
NF-κB: nuclear factor-κB transcription factor
FLA-PA: flagellin from Pseudomonas aeuruginosa.
