# Supplementary figures and images for: Flagellin From Pseudomonas Aeruginosa Stimulates ATB0,+ Transporter for Arginine and Neutral Amino Acids in Human Airway Epithelial Cells
Source: Front Immunol. 2021 Mar 25;12:641563. doi: 10.3389/fimmu.2021.641563 (PMC8029981; doi:10.3389/fimmu.2021.641563)

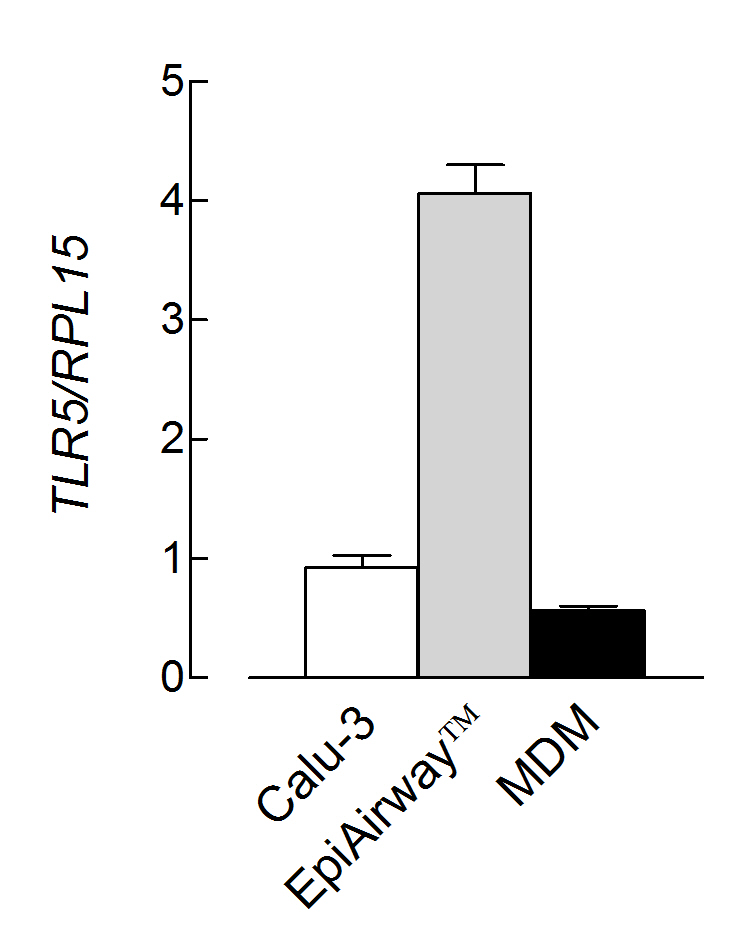

Supplement: Supplementary Figure 1 — The expression of toll-like receptor-5 (TLR5) was measured by means of RT-qPCR analysis and shown after normalization for that of the housekeeping gene (RPL15). Data of the three different cultures are presented as mean ± SEM. Monocyte-derived macrophages (MDM) were obtained from human monocytes after 5 day differentiation in the presence of 50 ng/ml GM-CSF. [file Image_1.JPEG]
